# Off-hours presentation is associated with short-term mortality but not with long-term mortality in patients with ST-segment elevation myocardial infarction: A meta-analysis

**DOI:** 10.1371/journal.pone.0189572

**Published:** 2017-12-28

**Authors:** Bingjian Wang, Yanchun Zhang, Xiaobing Wang, Tingting Hu, Ju Li, Jin Geng

**Affiliations:** 1 Department of Cardiology, Huai’an First People’s Hospital, Nanjing Medical University, Huai’an, Jiangsu, China; 2 Department of Cardiology, Huai’an Second People’s Hospital, the Affiliated Huai’an Hospital of Xuzhou Medical University, Huai’an, Jiangsu, China; 3 Department of Nephrology, Taizhou Second People's Hospital affiliated with Yangzhou University, Taizhou, Jiangsu, China; 4 Department of Rheumatology, Huai’an First People’s Hospital, Nanjing Medical University, Huai’an, Jiangsu, China; University of Tampere, FINLAND

## Abstract

**Background:**

The association between off-hours presentation and mortality in patients with ST-segment elevation myocardial infarction (STEMI) remains unclear. We performed a meta-analysis to assess the impact of off-hours presentation on short- and long-term mortality among STEMI patients.

**Methods:**

We searched PubMed, EMBASE, and the Cochrane Library from their inception to 10 July 2016. Studies were eligible if they evaluated the relationship of off-hours (weekend and/or night) presentation with short- and/or long-term mortality.

**Results:**

A total of 30 studies with 33 cohorts involving 192,658 STEMI patients were included. Off-hours presentation was associated with short-term mortality (odds ratio [OR] 1.07, 95% confidence interval [CI] 1.02–1.12, P = 0.004) but not with long-term mortality (OR 1.00, 95% CI 0.94–1.07, P = 0.979). No significant heterogeneity was observed. The outcomes remained the same after sensitivity analyses and trim and fill analyses. Subgroup analyses showed that STEMI patients undergoing primary percutaneous coronary intervention do not have a higher risk of short-term mortality (OR 1.061, 95% CI 0.993–1.151). In addition, higher mortality was observed only during hospitalization (OR 1.072, 95% CI 1.022–1.125), not at the 30-day, 1-year or long-term follow-ups.

**Conclusions:**

Off-hours presentation was associated with an increase in short-term mortality, but not long-term mortality, among STEMI patients. Clinical approaches to decrease short-term mortality regardless of the time of presentation should be evaluated in future studies.

## Introduction

A “weekend effect” was first described in 2001; it refers to the phenomenon in which patients with acute events who are admitted to hospitals on weekends have higher in-hospital mortality than those admitted on weekdays, which may be attributed to decreased staff levels[1]. Since then, many studies have evaluated the association of off-hours presentation (weekday nights and weekends) with short-term mortality among patients with acute myocardial infarction (AMI); however, their conclusions have been inconsistent[2–7]. Kostis reported that AMI patients admitted on weekends had higher 30-day mortality than those admitted on weekdays, but this disparity disappeared when invasive procedures were included into the regression model[3]. Similarly, Kumar also demonstrated a decreased mortality risk in patients admitted on weekends after additionally adjusting for cardiac catheterization[7]. These data suggested that the lower availability of cardiac intervention might lead to a difference in mortality between patients admitted during off-hours and those admitted during on-hours.

A recent meta-analysis showed an increased risk of short-term mortality in AMI patients who presented during off-hours[8]. However, conflicting outcomes of patients who presented with ST-segment elevation myocardial infarction (STEMI) during off-hours and those who presented during on-hours have been reported[9–13]. Patients who presented during off-hours were less likely to undergo invasive procedures[3,14,15], such as primary percutaneous coronary intervention (PPCI). In addition, only a few AMI patients (5–60%) underwent PPCI in previous studies[3,6,14–16]. We speculated that undergoing PPCI or not might be the reason for the controversial conclusions. Therefore, we conducted this meta-analysis of available data on the association between off-hours presentation and clinical outcomes in STEMI patients.

## Methods

The present study was conducted according to MOOSE (Meta-analysis Of Observational Studies in Epidemiology) recommendations[17], following a registered protocol in the PROSPERO database (CRD42016042845).

### Data sources and search strategy

We performed a comprehensive review in PubMed, EMBASE, and the Cochrane Library from database inception to 10 July 2016, without limitations on language and publication status. The following search terms were used: (“coronary artery disease” or “coronary heart disease” or “myocardial infarction” or “acute coronary syndrome” or “ST-segment elevation myocardial infarction” or “percutaneous coronary intervention”) and (“off hour” or “out of hour” or “after hour” or “weekend” or “time”). We also manually searched the references of included articles, relevant reviews and meta-analyses.

### Study selection

Two reviewers (X.W. and Y.Z) independently screened available studies according to the inclusion criteria. All discrepancies were resolved by discussion with a third reviewer (J.G.). The inclusion criteria were as follows: 1) all included studies had to compare short- and/or long-term mortality between patients who presented during off-hours and those who presented during on-hours; 2) studies evaluating long-term mortality followed up patients for at least 12 months; and 3) the enrolled patients were diagnosed with STEMI. We excluded abstracts and unpublished studies.

### Data extraction

Two reviewers (X.W. and Y.Z) independently recorded the study characteristics and the clinical and demographic information of the enrolled patients in each study; a third reviewer (J.G.) verified the information. We considered 30-day mortality as short-term mortality; in studies not reporting 30-day mortality, we used in-hospital mortality instead. We extracted mortality data as either the number of deaths, odds ratios (ORs) with 95% confidence intervals (CIs) or hazard ratios with CIs. We preferentially used adjusted outcomes from the meta-analyses; in studies without adjusted estimates, we used unadjusted estimates. We calculated unadjusted ORs using the number of deaths when they were unavailable. We contacted the authors for any missing or unclear data.

### Quality assessment

The Newcastle-Ottawa scale, which contains 9 terms in 3 domains (selection, comparability and outcome), was employed to assess the quality of the eligible studies[18]. Studies with 7 or more terms were considered to have low risk of bias. Two reviewers (X.W. and Y.Z) independently evaluated the quality of the included studies, and a third reviewer (J.G.) resolved discrepancies.

### Statistical analysis

We considered hazard ratios to approximate the relative estimate expressed in other studies using ORs, in accordance with previous meta-analyses[8]. Stata 12.0 was used to analyze the pooled effects with ORs and 95% CIs. Heterogeneity was assessed using the I^2^ test, with I^2^ values more than 50% suggesting significant heterogeneity[19]. We preferentially estimated the pooled effect using a fixed-effects model (inverse-variance method)[20]; if significant heterogeneity was identified, we used a random-effects model (DerSimonian and Larid method) instead[21]. Given considerable heterogeneity, we also performed a sensitivity analyses by excluding one study at one time to evaluate the contribution of each included study. Publication bias was estimated using Egger’s test and a funnel plot with the trim and fill method, which was also utilized to adjust for publication bias from potential unpublished studies[22,23].

We performed subgroup analyses according to region (North America versus Europe versus others), timing of presentation (admission versus arrival versus procedure), definition of off-hours (weekend and night versus weekend versus night), outcome adjustment (adjusted versus unadjusted) and percentage of patients who underwent PPCI (100% versus <100%). We also performed subgroup analyses by duration of follow-up (in-hospital versus 30-day and 1 year versus 1–5 years versus ≥5years) to evaluate the potential effect of follow-up time on mortality. We considered a 2-tailed p value less than 0.05 as statistically significant.

## Results

**Fig 1:**
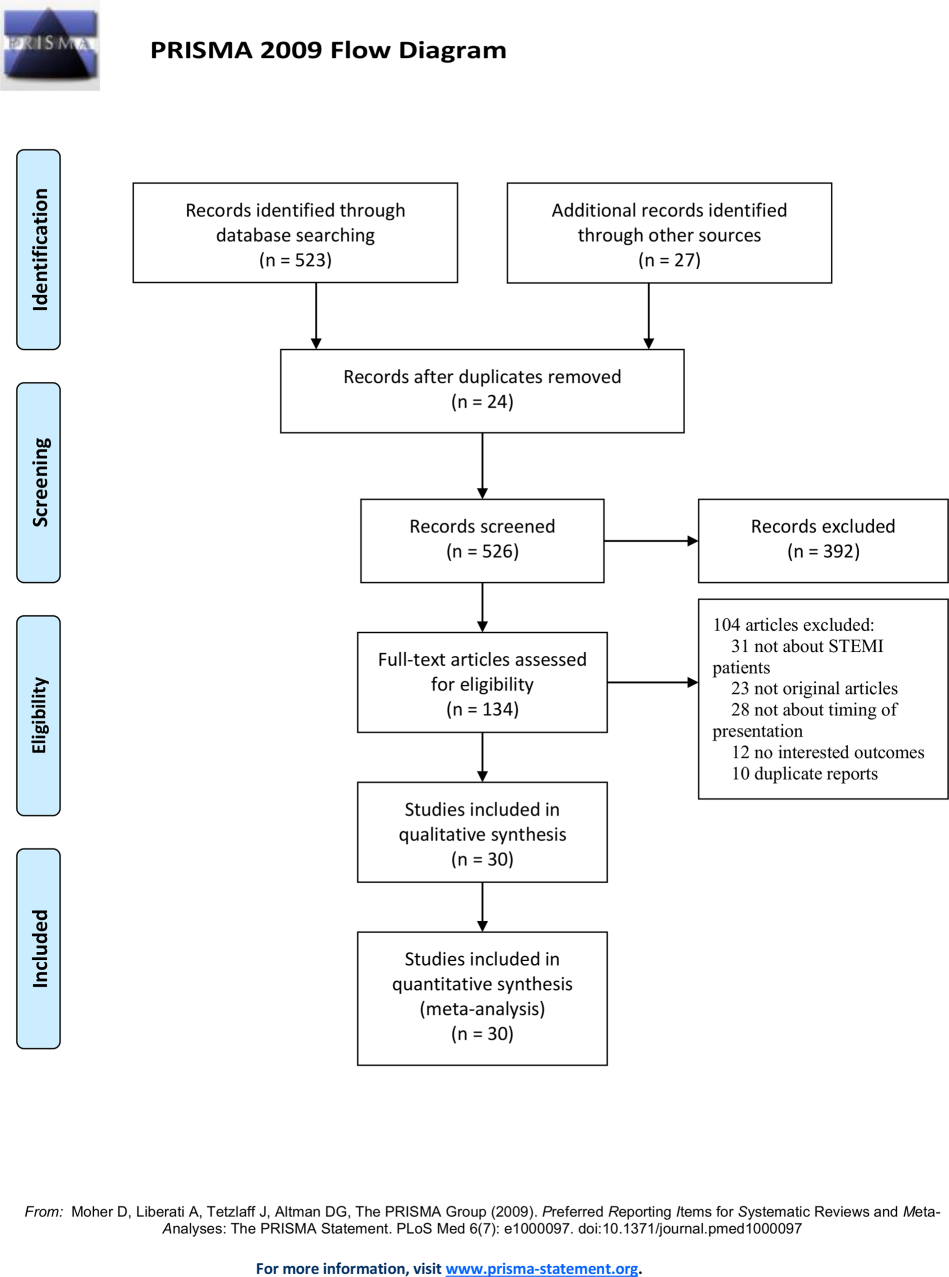
Flow diagram of the meta-analysis.

We included 30 studies comprising 192,658 STEMI patients after a two-step screening procedure (Fig 1). Of these, one study provided mortality data for three time intervals, and one reported results based on PPCI or fibrinolytic therapy[24,25]. Therefore, we evaluated 33 cohorts in the present meta-analysis. All the studies were published in English, except two, which were in Portuguese[26,27]. All patients were from a prospective clinical registry cohort, except two, which were from a retrospective cohort[9,28]. All studies retrospectively analyzed the collected data. Detailed characteristics of the eligible studies are presented in Table A in S1 File. Quality assessment of eligible studies according to the Newcastle-Ottawa scale is available in Table B in S1 File. The Newcastle-Ottawa scale score ranged from 7 to 9; thus, all studies were considered to have a low risk of bias. Demographic and clinical features are shown in Table 1. The STEMI patients who presented during off-hours were younger, predominantly male, had a higher prevalence of comorbidities and had worse cardiac function compared with those who presented during on-hours.

**Table 1:** Baseline characteristics of the included patients.

	Patients during off-hours	Patients during on-hours
Age (y), cohorts = 29, n = 158,068	61.4	62.8
Male (%), cohorts = 31, n = 171,233	67.7	66.4
LVEF (%), cohorts = 10, n = 16,551	46.5	47.8
Hypertension (%), cohorts = 29, n = 167,881	48.6	47.8
Smokers (%), cohorts = 28, n = 165,977	42.0	38.8
Hyperlipidemia (%), cohorts = 26, n = 163,550	39.0	38.5
Diabetes (%), cohorts = 31, n = 171,233	19.4	18.2
Previous MI (%), cohorts = 26, n = 156,943	16.5	16.1
Previous PCI/CABG (%), cohorts = 23, n = 150,759	8.7	7.7
Killip II-IV (%), cohorts = 8, n = 10,024	14.2	15.4
Cardiogenic shock (%), cohorts = 17, n = 150,160	4.0	3.6
β-blockers (%), cohorts = 7, n = 31,139	67.3	67.8
ACEIs/ARBs (%), cohorts = 5, n = 29,407	52.9	49.7
Statins (%), cohorts = 6, n = 30,154	56.7	51.9

LVEF = left ventricular ejection fraction; MI = myocardial infraction; PCI = percutaneous coronary intervention; CABG = coronary artery bypass graft; ACEI = angiotensin-converting enzyme inhibitors; and ARB = angiotensin receptor blockers.

### Short-term mortality

**Fig 2:**
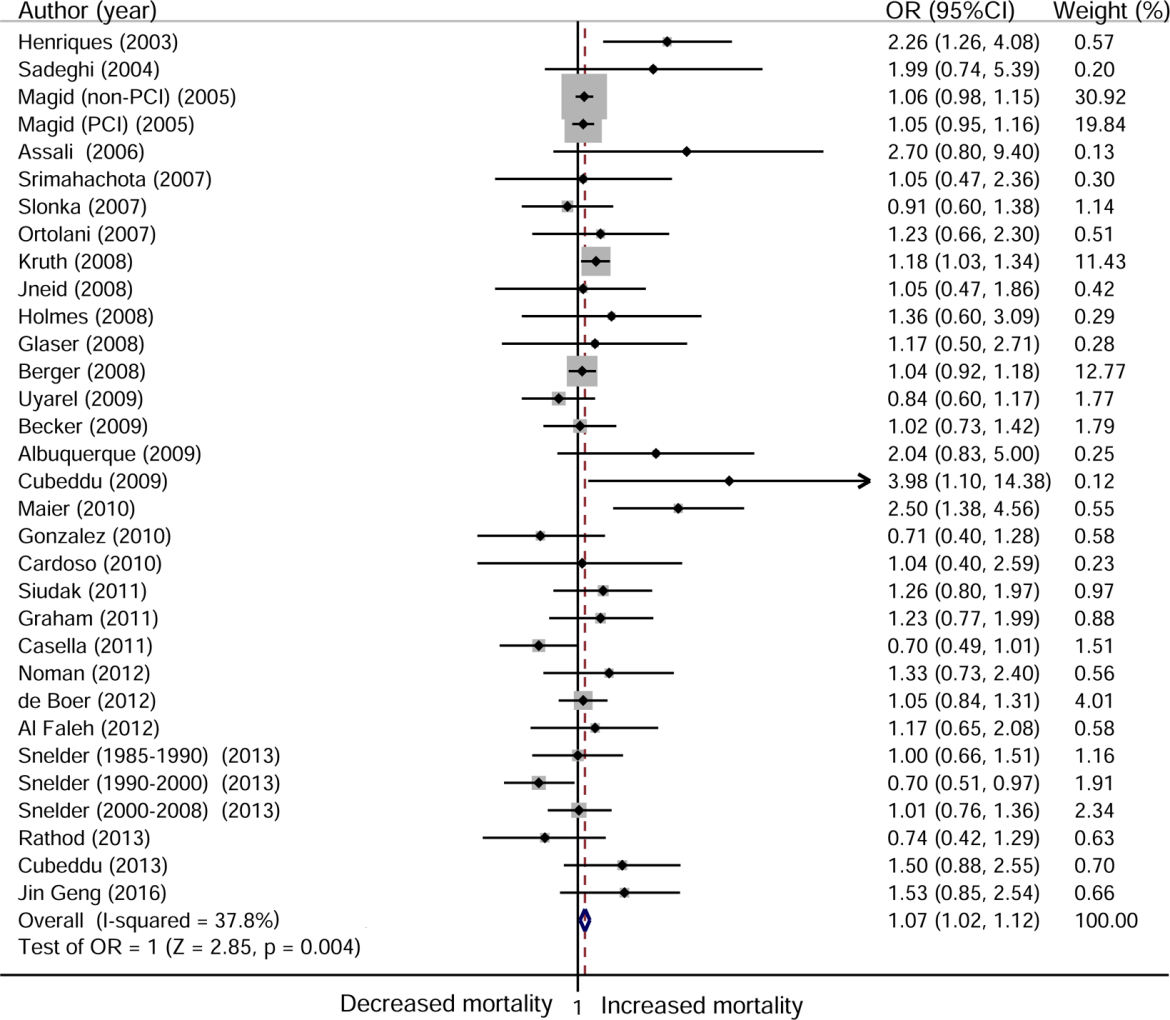
Forest plot for short-term mortality.

A total of 29 studies with 32 cohorts contributed data for 191,811 STEMI patients in the analysis of short-term mortality. Our meta-analysis revealed that patients who presented during off-hours had a significantly increased risk of short-term mortality compared to those who presented during on-hours, without significant heterogeneity among these studies (OR 1.07, 95% CI 1.02 to 1.12; I^2^ = 37.8%; Fig 2). We found no changes in the pooled estimate or the heterogeneity after the sensitivity analyses (Fig A in S1 File).

Off-hours presentation was associated with short-term mortality in cohorts from Europe (OR 1.081, 95% CI 1.008 to 1.159), in studies using admission time for definitions (OR 1.099, 95% CI 1.019 to 1.184), and in patients who presented during weekends and nights (OR 1.077, 95% CI 1.028 to 1.128). However, in the remaining subgroup analyses, no significant association was observed between off-hours presentation and early death. Outcome adjustment had no effect on the association of off-hours presentation with short-term mortality (adjusted OR 1.058, 95% CI 1.002 to 1.117; unadjusted OR 1.086, 95% CI 1.020 to 1.115). Interestingly, we found that off-hours presentation was associated with higher short-term mortality in cohorts with a low percentage (<100%) of patients undergoing PPCI (OR 1.084, 95% CI 1.016 to 1.126) but not in those with a high percentage of patients undergoing PPCI (OR 1.061, 95% CI 0.993 to 1.151). Moreover, patients who presented during off-hours had higher in-hospital mortality than those who presented during on-hours (OR 1.072, 95% CI 1.022 to 1.125); when this population was followed up for 30 days, however, there was no association between off-hours presentation and death (OR 1.037, 95% CI 0.923 to 1.165). No significant interaction was found among these subgroups (Table 2).

**Table 2:** Subgroup analyses of short-term mortality among patients with STEMI who presented during off-hours versus on-hours.

Subgroup	No of studies	OR (95% CI)	I^2^ (%)	P for interaction
Region				
North America	14	1.062 (0.999–1.128)	3.1	0.831
Europe	9	**1.081 (1.008–1.159)**	51.4	
Others*	9	1.022 (0.854–1.223)	44.8	
Timing of presentation measured				
Admission	13	**1.099 (1.019–1.184)**	61.7	0.492
Arrival	7	1.006 (0.844–1.144)	0	
Procedure	12	1.060 (0.997–1.127)	5.9	
Definition of off-hours				
Weekend and night	24	**1.077 (1.028–1.128)**	29	0.138
Weekend	3	0.855 (0.731–1.072)	36.9	
Night	5	1.121 (0.866–1.450)	61.3	
Adjustment				
Adjusted	20	**1.058 (1.002–1.117)**	50.4	0.585
Unadjusted	12	**1.086 (1.020–1.115)**	2.1	
Percentage of undergoing PPCI				
100%	22	1.061 (0.993–1.151)	45.6	0.898
<100%	10	**1.084 (1.016–1.126)**	19.8	
Type of mortality				
In-hospital mortality	21	**1.072 (1.022–1.125)**	34.2	0.599
30-day mortality	11	1.037 (0.923–1.165)	47.8	

OR = odds ratio; CI = confidence interval; STEMI = ST-segment elevation myocardial infarction; and PPCI = primary percutaneous coronary intervention.

*One study included STEMI patients from 11 countries.

**Fig 3:**
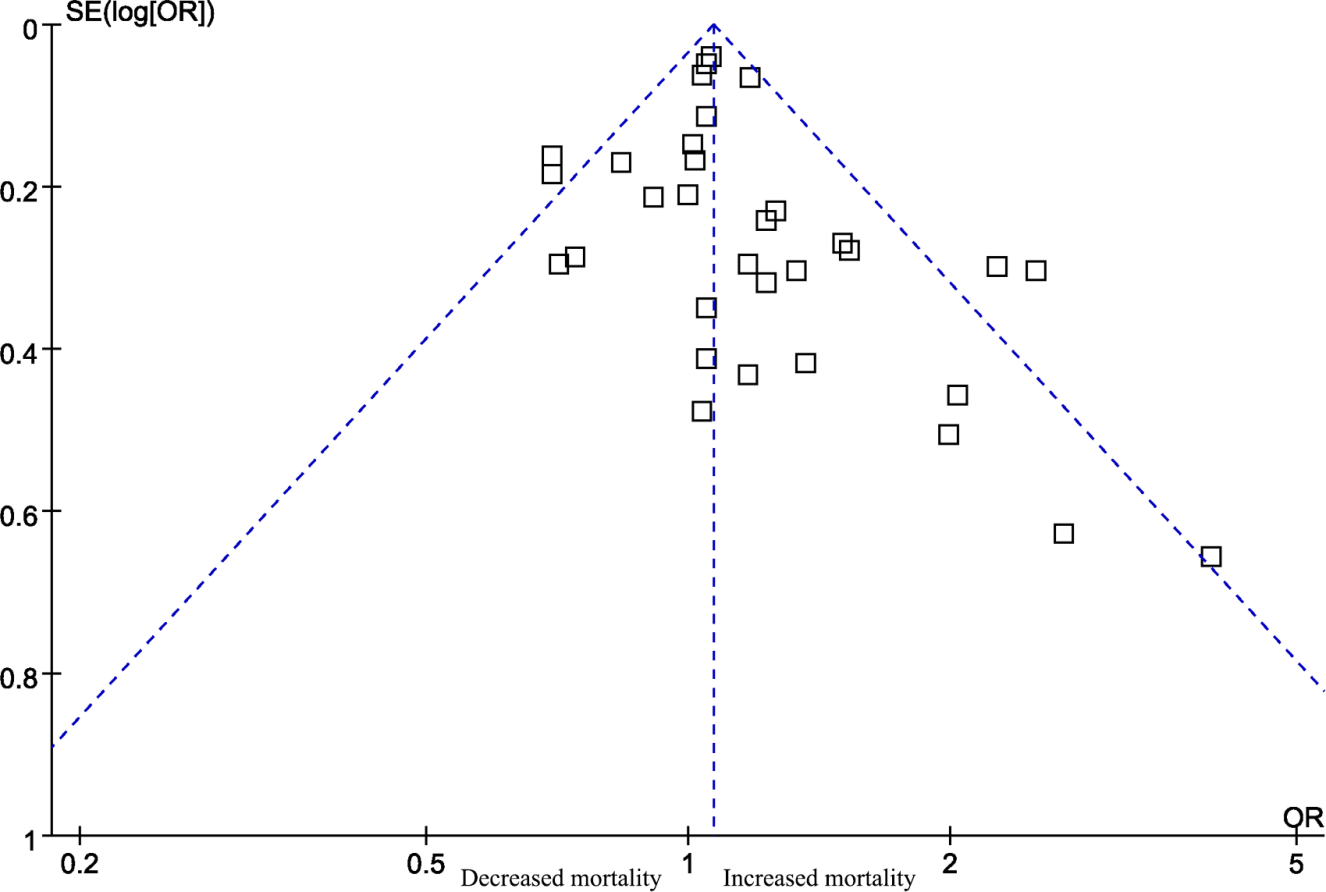
Funnel plot for short-term mortality.

Egger’s test revealed no statistically significant bias (P = 0.140); however, the funnel plot was asymmetric, indicating potential publication bias (Fig 3). We additionally conducted a trim and fill analysis and found that there might be 5 unpublished articles (Fig B in S1 File). Even after adding these 5 studies, the results regarding short-term mortality remained statistically significant (OR 1.052, 95% CI 1.007 to 1.100).

### Long-term mortality

**Fig 4:**
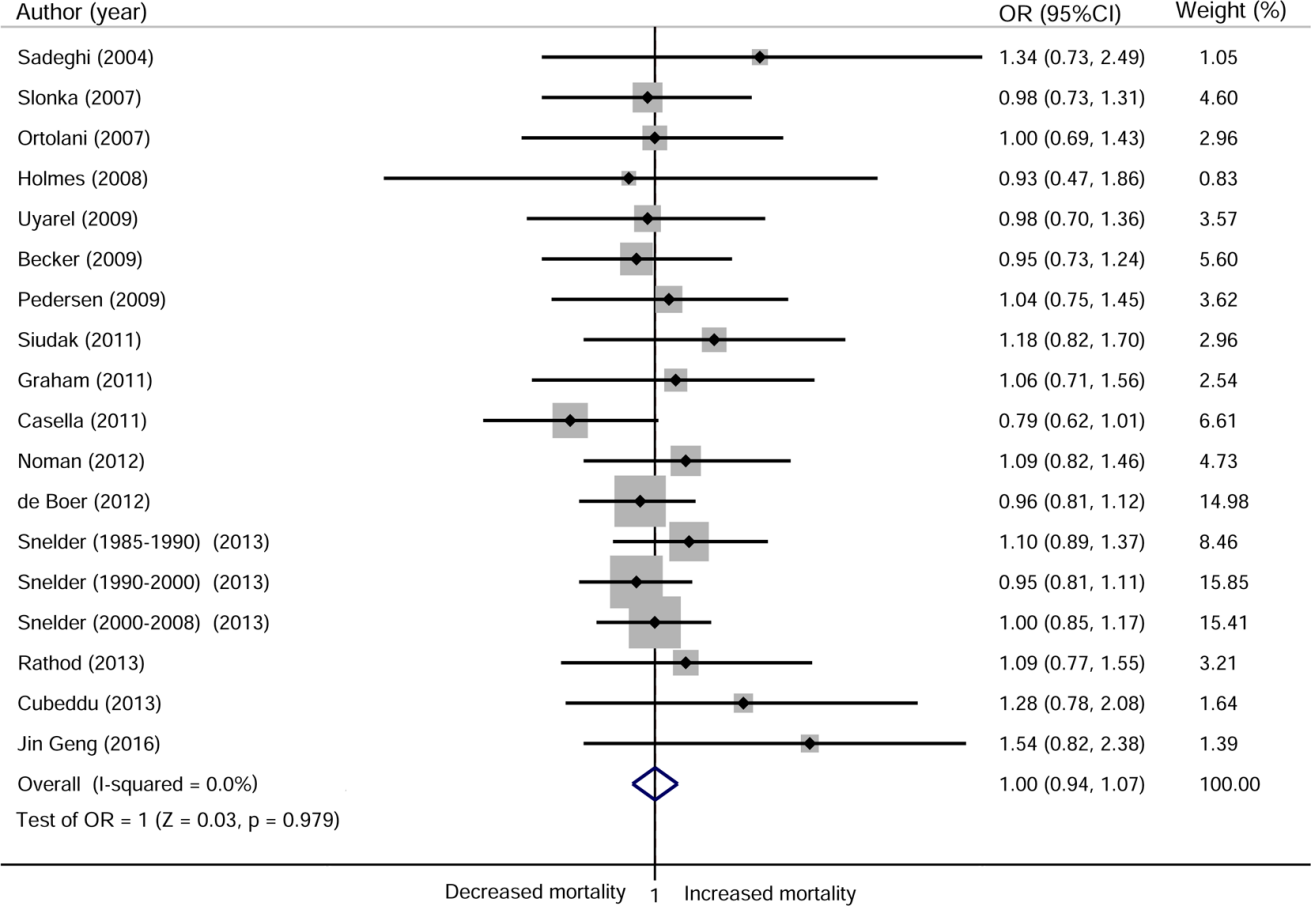
Forest plot for long-term mortality.

Sixteen studies comprising 38,184 subjects reported long-term mortality with a median follow-up of 2.5 years (range, 1 to 10 years; 18 cohorts). Two studies were followed up for 3 and 4 years, but as we only retrieved data from the 1-year follow-up, these studies were treated as 1-year follow-up studies[12,29]. There was no significant difference in long-term mortality between patients who presented during off-hours and those who presented during on-hours, and there was no evidence of heterogeneity (OR 1.00, 95% CI 0.94 to 1.07; I^2^ = 0%; Fig 4). Sensitivity analyses showed no alteration of the result after eliminating each study (Fig C in S1 File). Subgroup analyses showed that these findings were not affected by region, timing of presentation measured, definition of off-hours, outcome adjustment, percentage of patients undergoing PPCI and duration of follow-up; moreover, there were no significant interactions among these subgroups (Table 3).

**Table 3:** Subgroup analyses of long-term mortality among patients with STEMI who presented during off-hours versus on-hours.

Subgroup	No of studies	OR (95% CI)	I^2^ (%)	P for interaction
Region				
North America	3	1.093 (0.811–1.473)	0	0.724
Europe	11	1.004 (0.937–1.076)	0	
Others*	4	0.954 (0.802–1.133)	55.9	
Timing of presentation measured				
Admission	6	1.004 (0.920–1.096)	0	0.588
Arrival	4	1.117 (0.883–1.413)	0	
Procedure	8	0.978 (0.887–1.078)	0	
Definition of off-hours				
Weekend and night	14	1.003 (0.923–1.090)	0	0.991
Weekend	3	0.855 (0.731–1.072)	0	
Night	1	1.000 (0.905–1.104)	-	
Percentage of undergoing PPCI				
100%	13	0.997 (0.919–1.083)	0	0.897
<100%	5	1.006 (0.913–1.108)	0	
Duration of follow-up				
1 year	8	0.959 (0.866–1.062)	0	0.445
1~5 years	6	1.074 (0.932–1.237)	0	
≥5 years	4	1.006 (0.914–1.107)	0	
Adjustment				
Adjusted	13	1.000 (0.934–1.071)	0	0.961
Unadjusted	5	1.004 (0.858–1.175)	0	

OR = odds ratio; CI = confidence interval; STEMI = ST-segment elevation myocardial infarction; and PCI = primary percutaneous coronary intervention.

*One study included STEMI patients from 11 countries.

**Fig 5:**
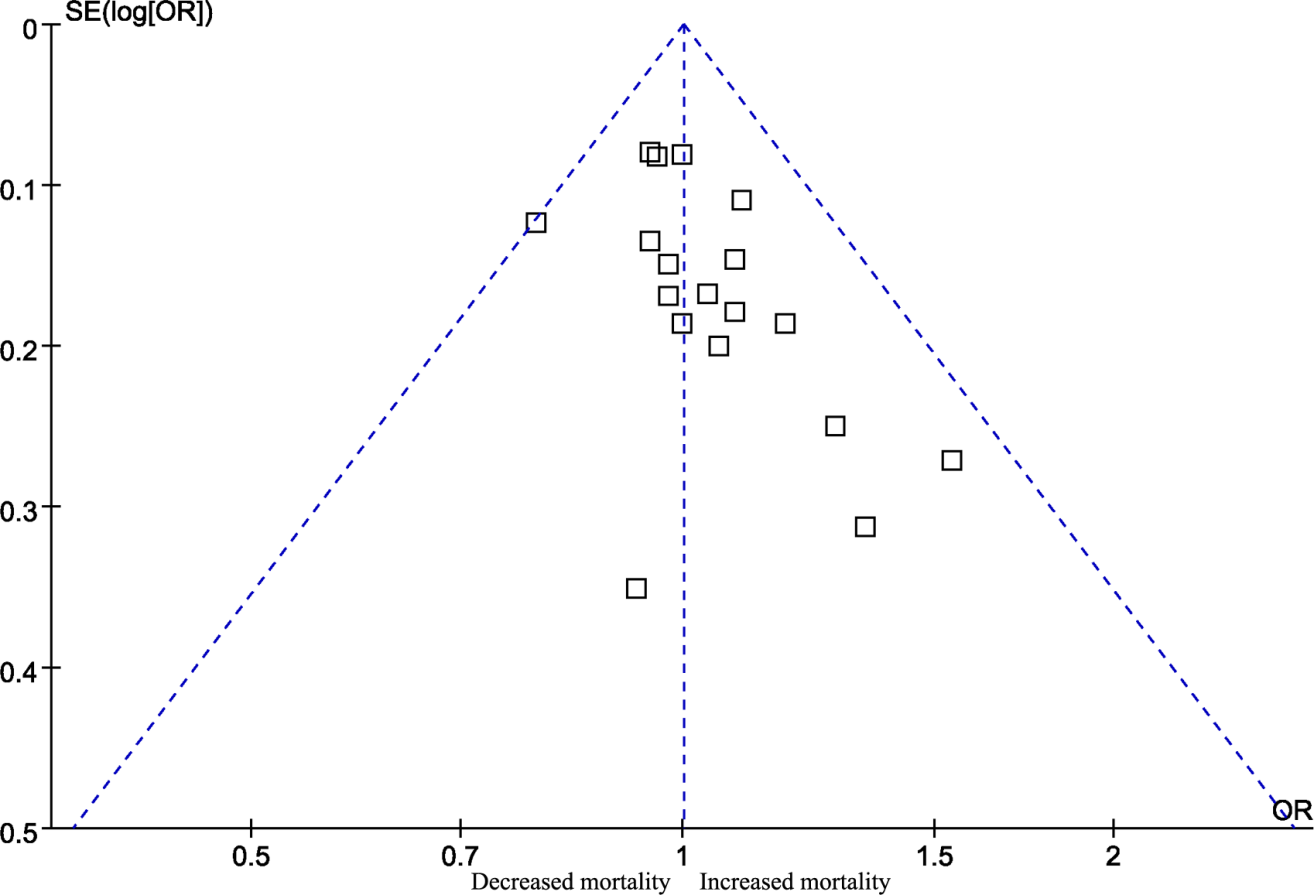
Funnel plot for long-term mortality.

Egger’s test indicated statistically significant bias (P = 0.028), and visual inspection of the funnel plot revealed that it may be asymmetric (Fig 5). The trim and fill method predicted that there may be 6 unpublished studies (Fig D in S1 File), although the difference remained nonsignificant after adjusting for these 6 studies (OR 0.966, 95% CI 0.912 to 1.023).

## Discussion

Using pooled data from more than 30 cohorts, we found that off-hours presentation was associated with an increased risk of mortality in STEMI patients. Higher mortality during off-hours was observed only during hospitalization, not in the 30-day, 1-year and even long-term follow-ups. In addition, the difference in short-term mortality was significant in Europe and among patients admitted on weekends and nights. Subgroup analyses indicated that PPCI might play a protective role in STEMI patients who presented during off-hours. Our findings of the main outcomes are strengthened by the facts that the sensitivity analyses and trim and fill methods did not alter the results and that no significant heterogeneity was observed.

A previous systematic review also concluded that patients with AMI who presented during off-hours had higher morality[8]. The present meta-analysis, however, differs from the previous systematic review as follows. First, we exclusively included studies that evaluated the “weekend effect” in STEMI patients and included 33 cohorts to assess the effect of off-hours presentation. Sorita analyzed mortality data from an AMI population, and the conclusion regarding STEMI patients was derived from one of the subgroup analyses among 25 cohorts[8]. Second, Sorita only provided in-hospital and 30-day mortality data, both of which indicated higher mortality among AMI patients who presented during off-hours. However, in our data, off-hours presentation was associated with in-hospital mortality, but not 30-day mortality among STEMI patients. Moreover, we added long-term mortality data from 18 cohorts and found no significant difference between STEMI patients who presented during off-hours and those who presented during on-hours. Third, based on our results, PPCI might be a protective factor for STEMI patients who presented during off-hours, which was similar to previous findings[3,7]. Finally, Sorita’s results might be limited by high heterogeneity, whereas in the present meta-analysis, we did not observe significant heterogeneity. In addition, we confirmed the high credibility of our main outcomes by performing a sensitivity analysis and a trim and fill analysis.

Several factors may contribute to the association between off-hours presentation and high short-term mortality. Off-hours presentation is associated with adverse baseline characteristics, such as a higher prevalence of cardiac risk factors and lower ejection fraction; previous studies, including ours, have also found that patients who presented during off-hours had more severe symptoms than those who presented during on-hours[16,28,30], which may partially explain the increased short-term mortality. Another potential contributor is lower availability of PPCI, according to the result of our subgroup analysis. Previous studies have demonstrated that patients who presented during off-hours were less likely to undergo invasive procedures, thus leading to worse outcomes[3,7,14]. In addition, the duration between arrival and revascularization is longer among patients who present during off-hours. STEMI patients may experience a relative 20% to 40% increase in short-term mortality due to a 30-minute delay in door-to-balloon (DTB) time[31,32]. Insufficient staffing may be another reason for the difference in morality. During off-hours, an on-call team is often required to provide prompt care, which may play a role in the low availability of intervention and prolonged DTB time, thus leading to higher mortality during off-hours[8].

Interestingly, we found that off-hours presentation had no influence on long-term mortality. We speculated that the reasons for higher short-term mortality had little effect on long-term death in STEMI patients presented during off-hours. STEMI patients always take medications that can improve prognosis after hospital discharge, which may alleviate the unfavorable effect of off-hours presentation during long-term follow-up. Moreover, prolonged DTB time did not significantly increase long-term mortality[33]. In-hospital mortality, however, increased significantly with increasing DTB time[31,32]. Our previous data indicated that STEMI patients with high thrombolysis in myocardial infarction risk scores had heightened long-term mortality during off-hours presentation, while those with low and moderate risk scores did not[28]. However, the majority of STEMI patients had low and moderate risk scores[28], which may be another reason why we could not detect a difference in long-term mortality between the off- and on-hours presentation groups.

Aldridge and colleagues examined the causal associations between weekend specialist intensity and admission mortality across the English National Health Service[34]. The intensity of the specialist at each hospital was defined as the number of specialist hours per ten emergency admissions during the daytime on Sunday and Wednesday. The investigators surveyed 15,537 clinicians from 115 acute hospitals to obtain the preliminary data. The specialist intensity on Sunday was half of that on Wednesday. There was a 10% increase in mortality among patients admitted on weekends. However, there was no significant association between specialist intensity and mortality. Given the low response rate from the staff survey (45%) and the lack of patient characteristics, this conclusion should be confirmed by future studies. In addition, patients who present during off-hours are often in more severe conditions[16,28,30], and additional experienced doctors and nurses may be required for prompt care. Black et al speculated that the severity of sick patients who present during off-hours, rather than the quality of hospital care, is the reason for the differences in mortality[35]. Expanding diagnostic services, improved discharge processes and increased staffing management, including doctors and nurses, may be viable approaches to reduce the adverse effects of off-hours presentation[36].

This meta-analysis has several limitations. First, the pooled data were derived from observational studies, and the baseline characteristics of the included patients differed for some confounding factors. We preferentially extracted adjusted outcomes for the meta-analyses; for studies with no adjusted estimates, we used unadjusted estimates. We also conducted subgroup analyses stratified by outcome adjustment and observed no alterations in our main outcomes. Second, we did not assess the differences in DTB time for STEMI patients, as prolonged DTB time has been reported in many studies[14,24,28,37–41] and verified by a previous meta-analysis[8]. Third, significant publication bias was present based on the Egger’s test and the funnel plot. However, our results were unchanged after adjusting for potential unpublished studies by the trim and fill method. The outcomes of the sensitivity analysis also improve the credibility of the present meta-analysis. Fourth, most of the data from the included cohorts were prospectively collected; however, the databases were not specifically designed to address this issue. Additional studies are required to confirm our results. Finally, we recently demonstrated an increased risk of long-term mortality in severe STEMI patients who presented during off-hours. However, data were not available to evaluate the association between off-hours presentation and mortality in this population.

## Conclusions

Current evidence indicates that off-hours presentation may be associated with higher short-term mortality, but not long-term mortality, among STEMI patients. PPCI likely reduces short-term mortality in this population. To reduce the unfavorable influences of the “weekend effect”, efforts should be made to improve the healthcare system to ensure similar outcomes for patients regardless of the time they present to the hospital.

## Supporting information

S1 PRISMA Checklist Preferred Reporting Items for Meta-Analysis (PRISMA) statement checklist. (DOC)

S1 File Supporting information. Fig A. Sensitivity analyses for short-term mortality. Fig B. Trim and fill analysis for short-term mortality (Square indicates unpublished studies, n = 5). Fig C. Sensitivity analyses for long-term mortality. Fig D. Trim and fill analysis for short-term mortality (Square indicates unpublished studies, n = 6). Table A. Characteristics of eligible studies. Table B. Quality assessment of eligible studies. Abbreviations: CI = confidence interval; OR = odds ratio.
(RAR)
